# Supplementary material for: Histone H3K9 Demethylase JMJD2B Plays a Role in LXRα-Dependent Lipogenesis
Source: Int J Mol Sci. 2020 Nov 5;21(21):8313. doi: 10.3390/ijms21218313 (PMC7664202; doi:10.3390/ijms21218313)
Supplement: Supplementary file 1 [file ijms-21-08313-s001.pdf]

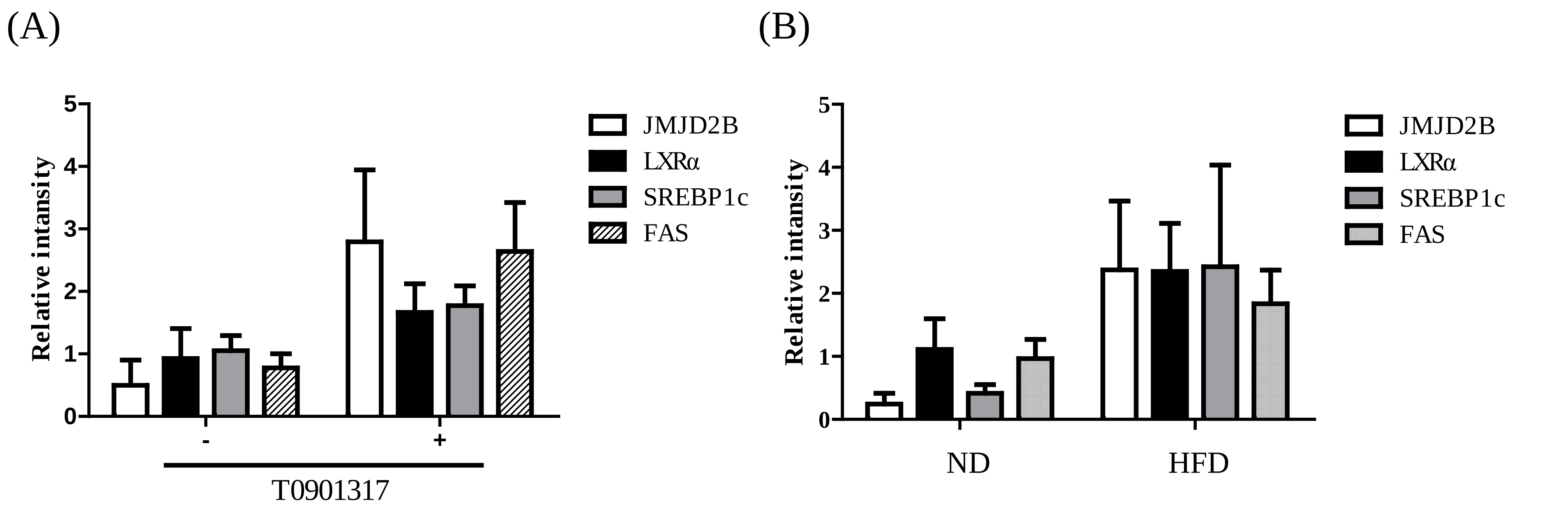

Supplementary Fig. S1. The bar graph of band density in figure 1I (A) and 2I (B)

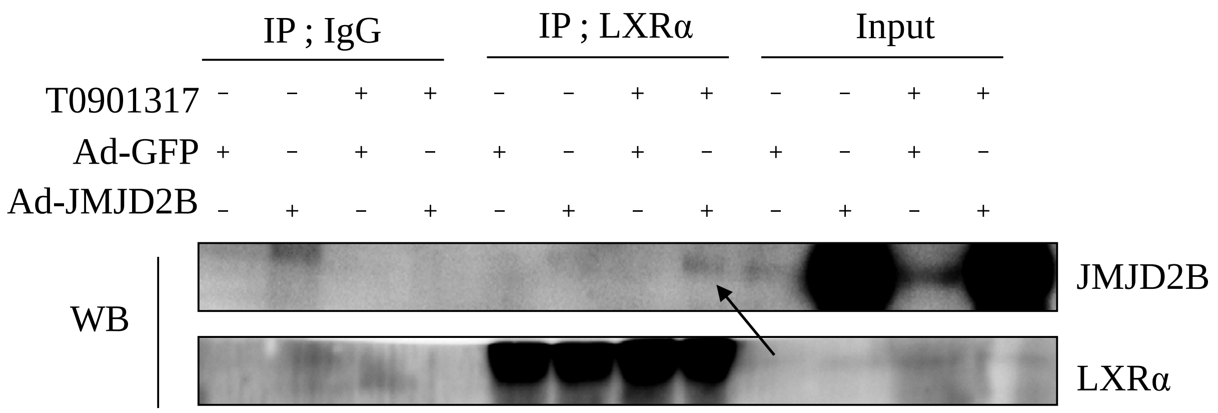

Supplementary Fig. S2. HepG2 cells were infected with Ad-GFP or Ad-JMJD2B and then incubated in the presence or absence of T09013178 for 24 h. Protein extracts were immunoprecipitated with LXRα antibody-agarose beads, and the interaction was detected with immunoblot analysis using JMJD2B antibody. Arrow indicates JMJD2B band detected in LXRα antibody-mediated immunoprecipitants.

Supplementary Table S1. Primer sequences used for real-time PCR

Species	Gene	Sequence (5’ → **3’)**
Human	JMJD2B	F: GGCCGGAGCTGCACACT<br>R: CGCGTCTTTGCACAGAGTAAGA
	LXRα	F: GCTCCTTTTCTGACCGGCTT<br>R: TGAATTCCACTTGCAGCCCT
	SREBP1c	F: CGG AGCCATGGATTGCACT<br>R: TAGGCCAGGGAAGTCACTG
	FAS	F: TCGTGGGCTACAGCATGGT<br>R: GCCCTCTGAAGTCGAAGAAGAA
	ACC	F: CTGTAGAAACCCGGACAGTAGAAC<br>R : GGTCAGCATACATCTCCATGTG
	SCD1	F: CACCACATTCTTCATTGATTGCA<br>R: ATGGCGGCCTTGGAGACT
	18S	F: CGGCTACCACATCCAAGGAA<br>R: GCTGGAATTACCGCGGCT
Mouse	JMJD2B	F: GGCCAAGATCATTCCACCCA<br>R: CCCACAGTCATGGCCTTCTT
	LXRα	F: ATCGCCTTGCTGAAGACCTCTG<br>R: GATGGGGTTGATGAACTCCACC
	SREBP1c	F: TGGATTGCACATTTGAAGACAT<br>R: GCCAGAGAAGCAGAAGAG
	FAS	F: AGG TGG TGA TAG CCG GTA TGT<br>R: TGG GTA ATC CAT AGA GCC CAG
	ACC	F: TGACAGACTGATCGCAGAGAAAG<br>R: TGGAGAGCCCCACACACA
	SCD1	F: CCGGAGACCCCTTAGATCGA<br>R: TAGCCTGTAAAAGATTTCTGCAAACC
	18S	F: CGGCTACCACATCCAAGGAA<br>R: CGGCTACCACATCCAAGGAA
